# Reply to “Beyond microbial carbon use efficiency”

**DOI:** 10.1093/nsr/nwae058

**Published:** 2024-02-07

**Authors:** Feng Tao, Johannes Lehmann, Ying-Ping Wang, Lifen Jiang, Bernhard Ahrens, Kostiantyn Viatkin, Stefano Manzoni, Benjamin Z Houlton, Yuanyuan Huang, Xiaomeng Huang, Yiqi Luo

**Affiliations:** Department of Ecology and Evolutionary Biology, Cornell University, USA; Soil and Crop Sciences Section, School of Integrative Plant Science, Cornell University, USA; CSIRO Environment, Australia; Soil and Crop Sciences Section, School of Integrative Plant Science, Cornell University, USA; Max Planck Institute for Biogeochemistry, Germany; Soil and Crop Sciences Section, School of Integrative Plant Science, Cornell University, USA; Department of Physical Geography and Bolin Centre for Climate Research, Stockholm University, Sweden; Department of Ecology and Evolutionary Biology, Cornell University, USA; Department of Global Development, Cornell University, USA; Key Laboratory of Ecosystem Network Observation and Modeling, Institute of Geographic Sciences and Natural Resources Research, Chinese Academy of Sciences, China; Department of Earth System Science, Ministry of Education Key Laboratory for Earth System Modelling, Institute for Global Change Studies, Tsinghua University, China; Soil and Crop Sciences Section, School of Integrative Plant Science, Cornell University, USA

In their commentary, Xiao *et al.* [1] cautioned that the conclusions on the critical role of microbial carbon use efficiency (CUE) in global soil organic carbon (SOC) storage in the paper by Tao *et al.* [2] might be too simplistic. They claimed that Tao *et al.*’s study lacked mechanistic consideration of SOC formation and excluded important data sets. Xiao *et al.* brought up important points, which can be largely reconciled with our findings by understanding the differences in expressing processes in empirical studies and in models.

Mechanistic understanding of complex processes from empirical research is usually translated into mathematical models with some level of simplification. For example, processes involved in SOC stabilization and persistence, as brought up by Xiao *et al.*, were considered using the model and evaluated together with microbial CUE for their relative importance to global SOC storage in Tao *et al.* [2]. The mechanisms for stabilizing necromass in soils with soil minerals are implicitly represented as the non-microbial carbon transfer using various chemical and physical processes (see carbon flows in Extended Data Fig. 3 in Tao *et al.* [2]). Parameter $a_{mSOC,MIC}$ represents the fraction of microbial necromass (subscript *MIC*) that is stabilized as mineral-associated SOC (subscript *mSOC*) via organo–mineral interactions (i.e. the *in vivo* pathway of stabilization; see Ref. [3]); parameter $a_{mSOC,LL}$ indicates the fraction of lignin litter (subscript *LL*) that is directly stabilized as SOC with minerals and without going through microbial processes (i.e. the *ex vivo* pathway of stabilization; see Supplementary Table 6 in Tao *et al.* [2]). The organic compounds associated with microbial products and necromass that Xiao *et al.* suggested to be stabilized against decomposition through various chemical and physical processes are expressed in the model by decomposition coefficients, *k_i_* (where subscript *i* refers to the *i*-th pool of carbon in a multipool ecosystem). The inverses of *k_i_* represent the residence time, which is a measure of persistence, of various organic compounds in soil. Tao *et al.* [2] compared the relative importance of non-microbial carbon transfer and decomposition coefficients with microbial CUE. The latter was found to be more important than the formers in determining SOC storage and its distributions at the global scale.

The dominant role of CUE in global SOC storage emerging from Bayesian inference by Tao *et al.* [2] does not imply that CUE is the only process to drive carbon storage, but it is likely a necessary process as soil might have fewer organo–mineral interactions without microbial metabolites. Our current understanding of stabilization mechanisms is highly fragmented from empirical research, which makes a fully process-based model representation very challenging. For instance, data syntheses have suggested statistically significant correlations between mineralogical or soil chemical variables (e.g. clay and silt fraction, short range-order iron, aluminum and exchangeable calcium) with soil carbon stocks at the continental scale [4–6]. However, mechanistic interpretations of the observed patterns at the global scale are still under development, leaving their mathematical representations in models at an early stage [7]. In the future, identifying new functional relationships and parameters that describe mineral stabilization in a mechanistic way is essential. Meanwhile, the inferred role of CUE in global SOC storage from our PROcess-guided deep learning and DAta-driven modelling (PRODA) approach should be further tested by more studies. We expect that not only other processes may be dominant in individual empirical studies, but that the relationship of CUE and SOC may vary among individual laboratory or site case studies.

We agree with Xiao *et al.* that causal relations between CUE and SOC need to be supported by more empirical evidence and mechanistic modeling studies. Tao *et al.* [2] showed both statistical (from the meta-analysis) and process-based (from the microbial model) evidence that microbial CUE promotes SOC storage at the global scale. First, Tao *et al.* [2] applied mixed-effects modeling to ensure the statistical rigor of the meta-analysis. The positive CUE–SOC relationship was robust after considering the influence of various predictors (e.g. temperature, soil depth, etc.) and their potential interactions (Extended Data Table 1 in Tao *et al.* [2]). Second, Tao *et al.* [2] investigated relationships among microbial CUE, microbial biomass and non-microbial biomass storage (i.e. the remaining amount of organic carbon after excluding microbial biomass; see Supplementary Table 2 in Tao *et al.* [2]).

**Table 1.** Unstandardized coefficients of the CUE–SOC relationship in the mixed-effects model including data from Malik *et al.* [8]. CUE, depth, mean annual temperature (MAT) and pH were set as the fixed effects to the logarithmic SOC content. The study source was set as the random effect. We set random intercepts with common slopes to test the CUE–SOC relationship. The total observation size ${{n}_{\\smallriptsize\textit{sample}}}$ = 295; the random effects size ${{n}_{\\smallriptsize\textit{study}}}$ = 17.

		Intercept	CUE	Depth	MAT	pH
$log10( {SOC} )\sim {CU\! E} + \textit{Depth} + MAT + pH + ( {1|Study\ \textit{Source}} )$ Variance explained by mixed model: 50%						
Fixed effects	Estimates	1.47	0.76	−0.019	0.012	−0.046
	Std. error	0.15	0.16	0.0034	0.0053	0.019
	*t*-value	10.02	4.82	−5.70	2.32	−2.50
	*P*	<0.0001	<0.0001	<0.0001	0.021	0.013
Random effects	Standard deviation	0.22	NA	NA	NA	NA

The results showed that a high CUE accompanied not only high microbial biomass carbon, but also high non-microbial biomass carbon. Third, the above findings in the meta-analysis were further verified by the results of the microbial model after data assimilation (Extended Data Table 2 and Supplementary Tables 3 and 4 in Tao *et al.* [2]). While the microbial model can theoretically generate positive, negative or null relationships between CUE and SOC, as noticed by Xiao *et al.*, Tao *et al.* [2] applied Bayesian data assimilation to identify the most probable regulatory pathway of CUE to SOC storage. That is, microbial partitioning of carbon toward microbial growth enhances SOC accumulation via microbial by-products and necromass. We acknowledge that this is inferred and not conclusive proof. The relationship between CUE and SOC might have complex interactions with other processes even though the result shown in Tao *et al.* [2] is an important step forward in mechanistically understanding SOC formation at the global scale and identifying what needs to be investigated in the future.

We greatly appreciate the point made by Xiao *et al.* that more data on microbial growth and respiration, especially from tropical and arid regions, are needed to avoid biased analyses. Our results support a positive relationship between microbial CUE and SOC storage based on two distinct lines of evidence. While the meta-analysis based on 132 data points showed an emerging positive CUE–SOC relationship across regions, the PRODA analysis of 57 267 globally distributed vertical SOC profiles supported this finding at the global scale and, thus, avoided potential regional biases in the meta-analysis. Nonetheless, we welcome any more field-measured microbial CUE and SOC data to further test the CUE–SOC relationship.

We thank Xiao *et al.* for bringing up the point that soil pH may alter the CUE–SOC relationship as shown in Malik *et al.* [8]. Including the data from Malik *et al.* [8] and considering pH as a fixed effect in the meta-analysis do not influence the overall positive CUE–SOC relationship (Table 1). Similarly, by using the microbial model data assimilation results, the Supplementary Table 3 of Tao *et al.* [2] further showed that other variables, such as bulk density, cation exchange capacity, clay content and net primary productivity, do not influence the positive CUE–SOC relationship across the globe. In their Fig. 2, Xiao *et al.* used a linear regression between CUE and SOC without considering any other factors, such as sampling depth, temperature and methodological differences across studies. These factors influence the CUE–SOC relationship and weaken the correlation. When discussing the relationship between two variables, accounting for potentially confounding factors is essential in a statistical analysis. Tao *et al.* [2] applied the mixed-effects models that accounted for the above factors to explore the relationship between microbial CUE and SOC. As a result, the positive CUE–SOC relationship explains the 55% variation in the observations.

Moreover, while conventional machine-learning methods are powerful tools for exploring spatial relationships between variables, exercising caution is necessary when interpreting their results. In addition to the concern about whether a small sample size is suitable for training a machine-learning model, the random forest used by Xiao *et al.* lacks uncertainty analyses to support their assertion. Meanwhile, using the increased prediction error of a random forest to quantify the importance of variables to SOC merely indicates statistical correlations. In contrast, Tao *et al.* [2] combined deep learning with a process-based model to quantify the relative importance of mechanism-related components to global SOC storage.

Establishing a globally causal link between CUE and SOC and evaluating the relative importance of soil carbon processes needs leverage of the potentials of empirical studies, process-based models and big data. We acknowledge that the model we used, as any models do, remains a simplified representation of real-world complexities of the soil system. Indeed, navigating sophisticated observations to a reasonable abstraction for useful predictions is part of the essence of modeling. Meanwhile, we agree with Xiao *et al.* that more sophisticated empirical measurements could improve our understanding of SOC formation. While models allow us to holistically evaluate soil as a system and the relative importance of their components, data from field measurements could provide direct evidence on key relationships in the soil carbon cycle. Tao *et al.* [2] developed the PRODA approach to effectively incorporate process-based models with big data to gain emerging understanding of global SOC storage. To our knowledge, it is presently a great challenge to experimentally evaluate the relative importance of the seven components of soil carbon dynamics in any laboratory and field studies. PRODA provides a common tool for both modelers and experimentalists to reconcile mechanistic understanding from empirical evidence and theoretical reasoning from modeling. New findings and relationships revealed using the PRODA approach will stimulate new experimental studies in laboratory and field, and improvement of soil carbon-cycling models.
